# Circulating tumor cell investigation in breast cancer patient-derived xenograft models by automated immunofluorescence staining, image acquisition, and single cell retrieval and analysis

**DOI:** 10.1186/s12885-019-5382-1

**Published:** 2019-03-12

**Authors:** Arturo B. Ramirez, Raksha Bhat, Debashish Sahay, Carmine De Angelis, Hariprasad Thangavel, Sina Hedayatpour, Lacey E. Dobrolecki, Agostina Nardone, Mario Giuliano, Chandandeep Nagi, Mothaffar Rimawi, C. Kent Osborne, Michael T. Lewis, Jackie L. Stilwell, Eric P. Kaldjian, Rachel Schiff, Meghana V. Trivedi

**Affiliations:** 1 RareCyte, Inc., Seattle, WA USA; 2 Department of Pharmacy Practice and Translational Research, University of Houston College of Pharmacy, Houston, TX USA; 3 Lester and Sue Smith Breast Center, Baylor College of Medicine, Houston, TX USA; 4 Department of Clinical Medicine and Surgery, University of Naples Federico II, Naples, Italy

**Keywords:** Breast cancer, Circulating tumor cells, Single-cell analysis, Patient-derived xenografts, Chemotherapy

## Abstract

**Background:**

Breast cancer patient-derived xenograft (BC-PDX) models represent a continuous and reproducible source of circulating tumor cells (CTCs) for studying their role in tumor biology and metastasis. We have previously shown the utility of BC-PDX models in the study of CTCs by immunohistochemistry (IHC) on serial paraffin sections and manual microscopic identification of cytokeratin-positive cells, a method that is both low-throughput and labor-intensive. We therefore aimed to identify and characterize CTCs from small volume mouse blood samples and examined its practical workflow in a study of BC-PDX mice treated with chemotherapy using an automated imaging platform, the AccuCyte®–CyteFinder® system.

**Methods:**

CTC analysis was conducted using blood from non-tumor bearing SCID/Beige mice spiked with human breast cancer cells, BC-PDX-bearing mice, and BC-PDX mice treated with vehicle or chemotherapeutic agent(s). After red blood cell lysis, nucleated cells were mixed with transfer solution, processed onto microscope slides, and stained by immunofluorescence. The CyteFinder automated scanning microscope was used to identify CTCs, defined as nucleated cells that were human cytokeratin-positive, and mouse CD45-negative. Disaggregated primary BC-PDX tumors and lung metastatic nodules were processed using the same immunostaining protocol. Collective expression of breast cancer cell surface markers (EpCAM, EGFR, and HER2) using a cocktail of target-specific antibodies was assessed. CTCs and disaggregated tumor cells were individually retrieved from slides using the CytePicker® module for sequence analysis of a BC-PDX tumor-specific *PIK3CA* mutation.

**Results:**

The recovery rate of human cancer cells spiked into murine blood was 83 ± 12%. CTC detection was not significantly different from the IHC method. One-third of CTCs did not stain positive for cell surface markers. A *PIK3CA* T1035A mutation present in a BC-PDX tumor was confirmed in isolated single CTCs and cells from dissociated metastatic nodules after whole genome amplification and sequencing. CTC evaluation could be simply implemented into a preclinical PDX therapeutic study setting with substantial improvements in workflow over the IHC method.

**Conclusions:**

Analysis of small volume blood samples from BC-PDX-bearing mice using the AccuCyte–CyteFinder system allows investigation of the role of CTCs in tumor biology and metastasis independent of surface marker expression.

**Electronic supplementary material:**

The online version of this article (10.1186/s12885-019-5382-1) contains supplementary material, which is available to authorized users.

## Background

Circulating tumor cells (CTCs) are found in the peripheral blood of most metastatic and some early-stage breast cancer (BC) patients [1]. CTCs are considered seeds of metastases since they detach from the tumor mass, enter the blood circulation, and can invade various distant sites of metastasis with a favorable environment for them to colonize and grow [2]. Beyond their proven role as a prognostic marker of survival in BC [3, 4], CTCs provide an easy-to-access population of malignant cells that may be used to interrogate tumor biology using phenotypic and genomic tools [5, 6]. The limitations of incorporating CTCs into routine clinical decision-making include their rarity and the paucity of evidence for CTC-guided therapy [7, 8].

In our previous research, we used BC patient-derived xenograft (BC-PDX) models for CTC research [9]. The PDX models are clinically relevant as a small fragment of patients’ tumor is transplanted directly into epithelium-free cleared mammary fat pads and allowed to grow in mice [10]. The PDX models retain the genomic, transcriptomic, and proteomic profile of the original tumor from patients and provide a constant and a renewable source of CTCs [9, 10]. We demonstrated that the presence of CTCs was highly correlated with that of bone marrow disseminated tumor cells, highlighting the clinical relevance of the PDX models as this observation is also seen in BC patients. We also observed the presence of CTC clusters in some of the PDX models, which was highly correlated with the metastatic potential of the models. Because of these results and our continued interest in utilizing PDX models for CTC research, we desired to find a process for CTC analysis that had less laborious workflow than our method, which required manual assessment of slides prepared from paraffin blocks of white blood cell (WBC) pellets stained for pan-cytokeratin (CK) by immunohistochemistry (IHC). This method precluded practical and efficient large-scale analysis of CTCs [9].

In searching for an alternative method for CTC isolation and detection, we found that immunocapture and size-exclusion systems have been successfully applied to preclinical mouse model analysis of CTCs [11, 12]. However, immuno-capture of CTCs is based on expression of EpCAM or other cell surface markers and has potential for omitting some CTCs that do not express these markers. We also excluded platforms that isolate CTCs from WBC based on size, since small CTCs may not be collected. In this study, we adapted and optimized the AccuCyte®–CyteFinder® system (RareCyte, Inc.) that combines density-based cell collection with direct cell imaging (“RareCyte platform”) [13, 14] to detect CTCs in small volume blood samples from BC-PDX models, sequence single CTCs identified and retrieved by the platform, and implement CTC analysis in a preclinical PDX therapeutic study.

## Methods

### Breast cancer PDX mouse models

Animal care for the mice bearing the BC-PDX tumors, as well as age- and gender-matched control mice, was in accordance with the NIH Guide for the Care and Use of Experimental Animals with approval from the Baylor College of Medicine Institutional Animal Care and Use Committee. Four to 5 weeks old female SCID/Beige mice purchased from Envigo and/or obtained from Dr. Michael Lewis’ laboratory breeding stock at Baylor College of Medicine were used to generate BC-PDX models. 3–6 animals were housed in large individual ventilated cages with ¼ inch pelleted cellulose bedding material and had access to chlorinated autoclaved water and 5V5R Purina diet ad libitum. Husbandry conditions such as pressure, temperature (68–72 °F), humidity (30–70%), lighting (12/12 light/dark cycle) were monitored 24 h.

The animals involved in the study had tumor volume and body weight measurements done twice weekly by one investigator for consistency. Mice weighed 14-17 g before tumor implantation surgery and 18-24 g at tumor harvest. The animals were transported to the laboratory in a new cage for blood collection during anesthesia and then euthanasia for tumor and tissue harvest. Isoflurane overdose (inhalation) was used as the preferred method of euthanasia when tumor reached 1000 mm^3^ due to the unavailability of CO2 tanks in the laboratory. Fragments from collected tumors were re-transplanted and passaged for up to 20 generations in additional mice. All the experimental procedures were performed during the day time in the laboratory. More details on the generation of PDX models and maintenance of PDX tumors have been previously described [10]. All PDX models used in this study have been characterized by our group for the presence of CTCs [9]. Three PDX models, BCM-4888, BCM-4272 and BCM-3887, were selected for the proposed studies because CTCs could be detected in relatively high numbers in these models [9].

### Processing of blood samples collected from BC-PDX mice

For blood collection, isoflurane inhalation was administered to mice placed on dissecting pad with their noses inside a 50 ml conical tubes containing cotton balls soaked in isoflurane. Induction of anesthesia was confirmed by absence of withdrawal reflex to toe pinch. Blood (400–600 μL) was collected from the inferior vena cava of control mice without the tumors as well as BC-PDX-bearing mice, as described previously [9]. The mice were immediately euthanized by cervical disarticulation followed by a secondary method of verification by bilateral opening of the thorax. WBCs were isolated by lysing red blood cells (RBCs) using RBC lysis buffer (eBioscience, #00–4333-57), and then washing and pelleting the remaining cells by centrifugation. For determining the CTC recovery rate, approximately 500 BT474 breast cancer cells (estimated by hemocytometer counting) were spiked into the isolated WBCs. For IHC analysis, the WBCs were processed as described before into paraffin blocks [9]. For RareCyte platform analysis, the WBCs were mixed with AccuCyte® transfer solution (1:4 volume ratio; 50:200 μl), incubated 10 min at room temperature, and the mixture was spread onto a total of 3 slides (80–90 μl/slide) using the CyteSpreader® device [13]. The slides were dried for 30 min at room temperature and shipped frozen to the RareCyte laboratory. The slides were fixed using 10% neutral buffered formalin for 1 h followed by neutralization with 1 M Tris pH 8.0 for 5–10 min before staining and analysis.

### CTC detection and retrieval

The formalin-fixed paraffin-embedded WBC pellets were sectioned, and nucleated CTCs were detected using anti-human pan-cytokeratin (CK, Clone AE1/AE3, Dako, Carpinteria, CA, USA) as described before [9]. CTCs were identified as hematoxylin-positive pan-CK+ cells. Cells on the slides processed with AccuCyte were labeled by multicolor immunofluorescence (IF) using the Ventana Discovery automated slide stainer. Slides were stained with DAPI (to mark nuclei), anti-human CK (AE1/AE3, eBioscience; C11, Biolegend), anti-mouse CD45 (30F11, Biolegend) cells, and a cocktail of antibodies against human cell surface markers [EpCAM (9C4, Biolegend), EGFR (EP38Y, Abcam), and HER2 (24D2, Biolegend)]. Stained slides were imaged by the CyteFinder® multi-channel scanning fluorescence microscope [13]. CyteMapper® software analyzed the scans and identified candidate cells that were presented to the reviewer for confirmation of CTC identity. Two independent reviewers identified CTCs from the scans and inconsistencies were resolved by consensus. CTCs were identified as DAPI+, human pan-CK+ and mouse CD45- cells. Individual CTCs were retrieved from slide by the CytePicker® module, which is integrated into CyteFinder as described previously, and placed into PCR tubes [13].

### Processing of mammary tumors and lung metastatic nodules

Tumors were isolated from PDX-bearing mice and weighed. The lung nodules were resected after the perfusion of the lungs using PBS. The primary tumors/lung nodules to be processed were submerged in PBS and kept on ice. About 10 ml of digestion medium per gram of tissue to be digested was added to the tumor sample (Digestion medium – ultra pure Collagenase III, 200–250 units/ml) as previously described [15]. The tumor was minced in digestion medium in 35 mm dishes, and then the digested suspension was incubated at 37 °C for 2–3 h in a 1.5 ml Eppendorf tube with frequent mixing during the incubation. The single cell suspension was filtered through 70 μm nylon mesh (cell strainers) and mixed with transfer solution (in 1:4 ratio) before being spread onto a slide mounted on CyteSpreader as described before [13]. The same fixation and staining protocols as above were followed for the detection of tumor cells.

### Single cell analysis of *PIK3CA* mutation

The BCM-4888 PDX model, containing a *PIK3CA* mutation T1035A, was used for single cell mutation analysis. Whole genome amplification (WGA) was conducted on single isolated CTCs, or disaggregated cells from the primary tumor or lung metastasis, using the PicoPLEX® WGA kit [Rubicon Genomics, #R300381] according to manufacturer’s specifications. WGA reaction products that yielded at least 1 μg DNA were used for *PIK3CA* gene sequencing. Approximately 1 μL of the WGA reaction product was used for amplification of the *PIK3CA* gene that encodes the region of the protein containing the T1035A mutation. Nested PCR primers were designed from the NCBI human reference genomic sequence and amplified using chr3:179203356+179204175 for the outer primers (5′- TCTTGTGCTTCAACGTAAATCC -3′ and 5′- GCTGGTGAAGCAGTACCTCAT -3′) and chr3:179203570+179203916 for the inner primers (5′- GAGGATGCCCAATTTGATGT -3′ and 5′- CGGAGATTTGGATGTTCTCC -3′) using Primer3 software [16, 17]. The amplicon generated from the outer primer set was 820 bp and from the inner primer set was 347 bp. The WGA product (~ 1 μL) was transferred into a PCR tube with 2X PCR reaction mix (New England Biolabs, Ipswich, MA, USA), 0.5 μM of each primer, and water was mixed and placed into a thermal cycler (Thermo Fisher Scientific). Thermal cycling conditions were as follows: (1) incubation at 94 °C for 7 min, (2) 30 cycles of 94 °C for 30 s, 60 °C for 30 s and 72 °C for 30 s, (3) final extension at 72 °C for 7 min. Samples were held at 4 °C until they were analyzed by gel electrophoresis. After PCR, the presence of the 347 bp amplicon was confirmed by loading a portion of the reaction onto a 2% agarose gel and staining with SYBR® safe (Invitrogen) and comparing its migration to a DNA size standard. The resulting amplicon was purified from primers using the DNA Clean & Concentrator (Zymo Research, Irvine, CA, USA) according to manufacturer’s instructions. Approximately 1 ng of amplicon was mixed with sequencing primer (inner PCR primers) and BigDye® Terminator sequencing reactions (Life Technologies) were performed according to manufacturer’s directions. Reactions were run on a 3730XL DNA Analyzer (ThermoFisher Scientific). Sequences were analyzed using Sequence Scanner software (Applied Biosystems) for the presence of the nucleotide mutation T1035A.

### Evaluation of CTCs after various chemotherapy regimens using triple-negative BC-PDX models

In order to incorporate CTC analysis as a part of preclinical trial using various chemotherapy regimens, we evaluated CTCs in mice from the BCM-4272 and BCM-3887 models. The tumors were allowed to reach ~ 200 mm^3^ size before the mice were randomized to receive vehicle, docetaxel 20 or 30 mg/kg, carboplatin 50 mg/kg, or the combination of both drugs (*N* = 3–5 in each group). The drugs or vehicle were administered via intraperitoneal route every week for a total of 4 weeks. Body weight and tumor volume were assessed by one investigator twice a week. The animal groups were treated and evaluated randomly, not in any specific order. The animals with residual tumors were euthanized to harvest tumors and blood for CTC evaluation at the end of the 4-week treatment period. The investigators involved in processing blood and assessing CTCs were not blinded to the treatment arms but were blinded to tumor measurement data. The animals with complete tumor response were followed for assessment of recurrence; however, the number was typically only 1–2 per group and therefore deemed insufficient to be included in the evaluation.

## Results

### Spike-in breast cancer cell recovery rate

**Fig. 1:**
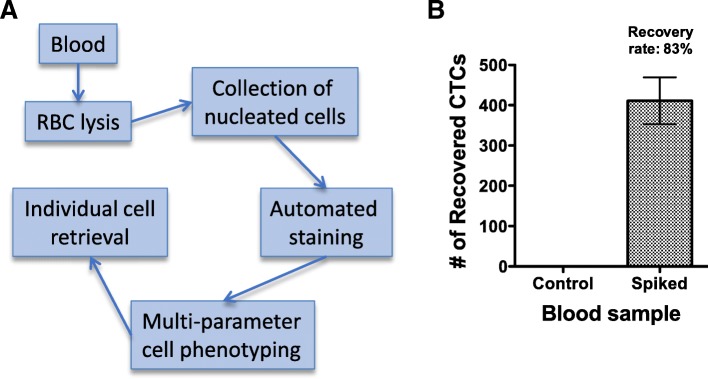
CTC detection and recovery using modified RareCyte method. **a**. Workflow using modified AccuCyte sample processing developed for mouse blood. This involved RBC lysis to isolate nucleated cells, which were then processed onto slides using the AccuCyte method [13]. **b**. CTC recovery rate. The tumor cell recovery rate was 83% in the blood samples from non-tumor bearing mice spiked with ~ 500 BT474 cells (*N* = 3). No CTCs were found in control blood samples not spiked with BT474 cells

A modified AccuCyte collection and processing protocol was developed for mouse blood (Fig. 1a). This incorporated RBC lysis to isolate nucleated cells, which were then processed to slides. Slides prepared from non-tumor bearing mouse blood samples spiked with human breast cancer BT474 cells were stained by immunofluorescence using an automated stainer, imaged by automated scanning microscopy, and analyzed by automated machine learning algorithms to identify and enumerate tumor cells as described in Methods [13]. The recovery rate of spiked-in cells was found to be 83 ± 12% (Fig. 1b). No tumor cells were identified in samples not spiked with cancer cells.

### CTC analysis in PDX models using RareCyte platform

**Fig. 2:**
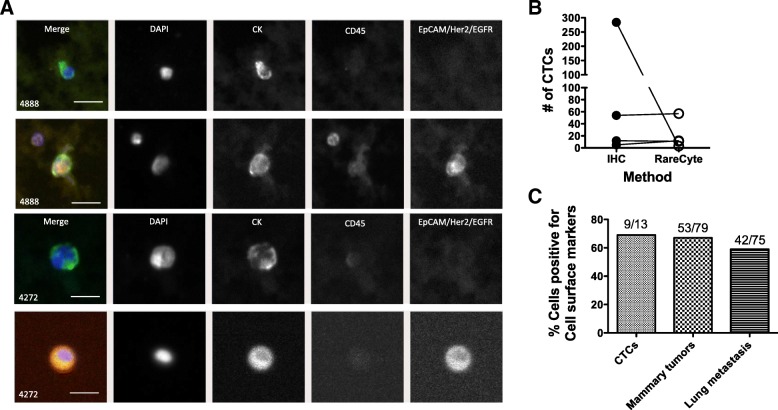
Comparison of modified RareCyte method to IHC method in detecting CTCs in BC-PDX-bearing mice. **a**. Representative immunofluorescence images of CTCs. Images show morphology of recovered CTCs. Column 1 is a composite/merged image; column 2 is DAPI staining of nuclei; column 3 is human cytokeratin staining; column 4 is mouse CD45 staining; and column 5 is staining with a cocktail of EpCAM, EGFR and HER2. (Scale bar equals approximately 20 μm) **b**. Comparison of IHC and RareCyte method for detecting CTCs in PDX models BCM-4272 and BCM-3887, which was not significantly different between IHC and RareCyte methods (*N* = 4; *P* ≥ 0.05, Paired t-test). **c**. Collective expression of cell surface markers (EpCAM, EGFR, HER2) on CTCs and BC-PDX tumor cells collected from mammary tumor and lung metastatic nodules in BCM-4888 model

We next applied the RareCyte platform CTC analysis method to PDX-bearing mouse blood samples and compared the results with our previously published IHC method [9]. Blood from two PDX-bearing mice was combined and equally divided into two tubes, one for each method, processed, stained and analyzed. The RareCyte platform identified CTCs from both the BCM-4888 and BCM-4272 models. Some CTCs were positive for both CK and at least one of the surface markers EpCAM, EGFR, and HER2; others were positive only for CK (Fig. 2a). Statistical analysis of the numbers of CTCs (Fig. 2b) yielded no significant difference between IHC and RareCyte platforms, although there was a discordance in one sample (*P* > 0.05, Paired Student’s t-test, *N* = 4).

Since the RareCyte platform allows preparation of cell suspensions for analysis, we made slide smears of dissociated mammary tumors and lung metastases taken from one PDX mouse (BCM-4888) to investigate the phenotypic comparison of CTCs with the solid tumor cells. 13 CTCs were identified in this mouse. Nine of the 13 CTCs (69%) stained positively with the cell surface marker cocktail, approximating the positivity of the mammary tumors (67%) and the lung metastatic nodules (59%) (*P* < 0.05, Chi-square analysis) (Fig. 2c). These results suggest that an immunocapture method for CTCs based on surface markers would have failed to identify about a third of the CTCs found in this animal.

### Single cell mutation analysis in CTCs

**Fig. 3:**
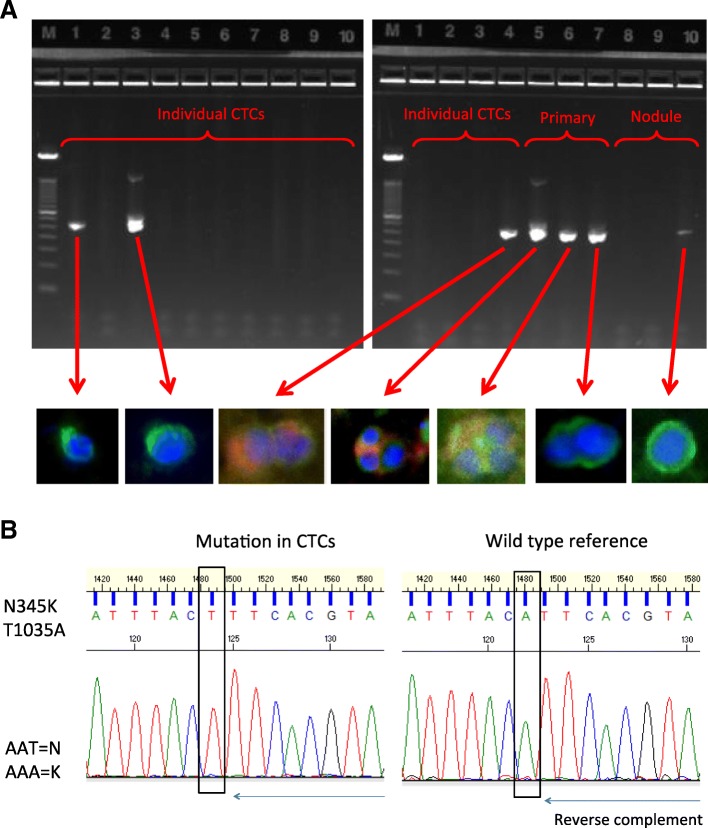
Analysis of *PIK3CA* T1035A mutation in CTCs, primary tumors, and lung metastases from BC-PDX model BCM-4888. **a** Whole genome amplification was conducted on single cells using Rubicon PicoPLEX® kit, followed by nested PCR using primers spanning the PIK3CA mutation. PCR products were run on an agarose gel to confirm the presence of an amplicon of the correct size. Images of the single cells picked and then used for the WGA are shown below the gel image (scale varies between cells). **b** Sanger sequencing was applied to the purified amplicons using internal primers to confirm presence of the T1035A mutation in the primary tumor cells, CTCs, and lung metastasis cells. A wild type reference derived from HeLa DNA was run in parallel. This is a representative image of the sequencing trace from the cells containing the mutated gene

Single cell analysis of CTCs may be used to investigate molecular heterogeneity of CTCs as well as to identify clones from a primary tumor that have capacity for metastasis. To investigate single cell mutational analysis, we tested whether a known *PIK3CA* mutation in the PDX model BCM-4888 [T1035A (N345K)] could be identified in individual CTCs, using disaggregated cells from the primary mammary tumor or lung metastasis as controls. CTCs identified as above were mechanically retrieved from slides and whole genome amplification (WGA) was performed. A PCR amplicon spanning the region with the mutation was successfully generated from the WGA product in 3 of 3 cells from the primary tumor, 1 of 3 cells from the lung nodule, and 3 of 13 single CTCs analyzed from the same mouse (Fig. 3a). All *PIK3CA* amplicons – from the primary tumor, CTCs and lung nodule – contained the T1035A mutation (Fig. 3b) supporting a common clonal origin of the PDX model cells. Lanes without bands in Fig. 3a are from single cells for which a *PIK3CA* amplicon was not generated; this may be due to non-uniform WGA leading to occurrence of “drop-out” regions, a currently recognized challenge when amplifying a single genome. Optimization of amplification methods is expected to improve efficiency of single cell sequence analysis.

### Incorporation of CTC analysis in chemotherapy-treated PDX models

**Fig. 4:**
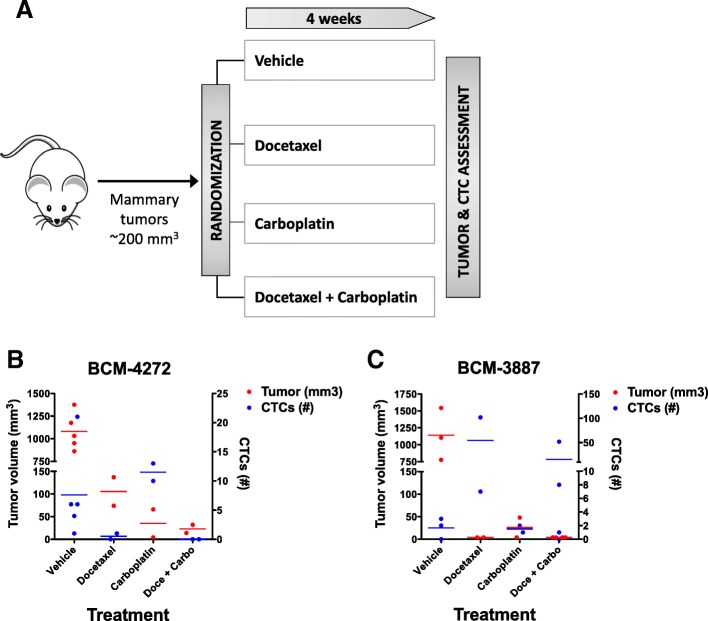
Preclinical chemotherapy treatment scheme and findings in PDX-bearing mice of BCM-4272 and BCM-3887. **a** Overall experimental scheme. Mice in two PDX models (BCM-4272 and BCM-3887) were randomized to receive either vehicle, docetaxel 20–30 mg/kg; carboplatin 50 mg/kg; or their combination. Intraperitoneal treatment was given every week for 4 weeks. Tumor volume and CTCs were evaluated at the end of 4-week treatment (*N* = 2–5 in each treatment group) in mice with residual disease for each PDX model. **b** and **c** Tumor volume and CTC numbers in mice treated with vehicle or various chemotherapy regimens for BCM-4272 and BCM-3887 models, respectively. Treatment with various chemotherapy regimens reduced tumor growth in both triple-negative BC-PDX models, BCM-4272 and BCM-3887 however CTCs persisted despite tumor reduction in some regimens

Finally, we aimed to incorporate CTC analysis using RareCyte platform as a part of practical pharmacotherapeutic studies in PDX models. In a small prototypic study, tumor-bearing mice from triple-negative BC-PDX models, BCM-4272 and BCM-3887 were randomly assigned to receive weekly treatment with vehicle, docetaxel, carboplatin or docetaxel + carboplatin; blood was collected at the end of the 4-week treatment for CTC analysis (Fig. 4a). No significant adverse events requiring dose delay or reduction were noted. The blood processing method was simple to implement at a scale of approximately 12 animals per processing time point. Slide banking and automated immunostaining allowed batches of 12 to 30 slides to be stained simultaneously in less than 2 h. Time for scanning, image analysis and review was approximately 15–20 min per slide.

The tumor volumes and number of CTCs in the various treatment groups are illustrated in Fig. 4b and c, respectively. CTCs were detectable in mice in all arms of both PDX model experiments, except for the docetaxel + carboplatin arm of BCM-4272 (Fig. 4b and c). In both models, treatment with the chemotherapy regimens reduced tumor growth, however CTCs persisted after tumor reduction in some regimens, and no clear association was present between CTC number and primary tumor volume response. It should be noted that this study was not designed to test whether such an association exists.

## Discussion

In this study, we report adaptation of the RareCyte AccuCyte–CyteFinder system as a platform to evaluate CTCs in BC-PDX models. The 83% recovery rate for spiked in tumor cells is similar to that reported using the original method in previous studies using cancer cells spiked into human blood [13]. Furthermore, we find that the RareCyte platform is comparable to our previously published IHC method [9] in detecting CTCs, with workflow advantages of sample processing, CTC identification, and semi-automated retrieval of individual CTCs for molecular analysis. These findings indicate that this platform can be used to collect, identify and characterize CTCs in blood from mouse models in preclinical pharmacotherapy studies.

The volume of blood used in this study, ~ 500 μL, can readily be obtained at a terminal collection. There is interest and considerable value in being able to monitor CTCs during the course of preclinical therapeutic studies in mouse models by serial blood collection to monitor treatment response, identify drug resistant tumor cell population, and characterize mechanisms of resistance. This requires volumes of 100 μL or less. To assess linearity of very small volume sample CTC analysis, a two-fold dilution study was performed with spike-in cells using volumes from 1000 down to 62.5 μL. The results were highly linear (R^2^ = 0. 97, Additional file 1: Figure S1), suggesting that using smaller volumes is achievable.

Our preclinical pharmacotherapy trial was designed to assess the applicability of the mouse CTC analysis method, and thus did not have adequate sample size to statistically assess whether CTCs were prognostic of cancer progression. However, the method has been applied in preclinical studies that revealed statistically significant associations. A recently presented orthotopic breast cancer xenograft study of a MAP kinase-activated protein kinase 2 (MK2) inhibitor reported significant dose-dependent reduction in CTC count and increased number of CTC-free animals following treatment [18]. CTC number was also significantly correlated with the number of lung metastases, leading the authors to suggest that CTCs may be used to monitor the effectiveness of MK2-inhibitors to block metastasis in certain tumors. It should be noted that sample volumes in this study were between 50 and 200 μL, lending further support to the use of the platform for longitudinal analysis in mice. In another study investigating PDX models from various tumor types, CTCs were observed in 9 of 9 models and the total number of CTCs and CTC clusters was positively correlated with the frequency and size of lung metastasis [19].

In addition to collection and identification of CTCs for the monitoring of response to therapy in mouse models, it is of high research interest to be able to characterize CTCs by phenotype and genotype. We employed a cocktail of antibodies to cell surface markers EpCAM, EGFR, and HER2 in a biomarker channel independent of the marker (cytokeratin) used to identify the CTCs. Here, we also used a cocktail of cytokeratin antibodies (clone AE1/AE3 and clone C-11) to increase the coverage for pan-CKs, which are able to detect both epithelial CTCs and those undergoing EMT [20]. The purpose was to address the question of whether the method we have developed does in fact identify CTCs that would not be collected using a cocktail surface capture technology, and thus provide a more comprehensive CTC identification platform. Staining with the cocktail was absent on nearly a third of CTCs in one of the PDX models, suggesting heterogeneity in the expression of epithelial surface proteins and highlighting the fundamental constraint of technologies that rely on expression of surface markers: only CTCs that express the surface proteins can be isolated [21–23]. Other recent reports have similarly underscored the drawbacks of surface protein capture methods for comprehensive CTC collection in preclinical models [11]. In future studies, investigation of the cell surface markers independently should be considered to provide the depth of analysis that, to our knowledge, has not been reported on individual CTCs.

Analysis of CTCs for assessment of the presence of tumor-specific mutations in patient samples has been established [24–26]. By isolating individual CTCs after slide-based visualization, we were able to confirm presence of a known *PIK3CA* mutation, and thus demonstrate that single-cell sequence analysis can be performed in CTCs collected from PDX models using this approach. Recently, ctDNA has gained popularity for monitoring tumor-specific mutations in the blood and prediction of tumor recurrence due to the ease of analysis. However, ctDNA-based mutation analysis has several limitations. For example, ctDNA may not find mutations that are present at low allelic frequencies. Furthermore, the likely source of ctDNA is the apoptotic or necrotic tumor portion and not the actively dividing cancer cells, which may make it less relevant for investigating resistant clones that survive during ongoing treatment, which are by definition, resistant to therapy. It is also increasingly theorized that the evaluation of mutations in CTCs in addition to ctDNA will provide a better and complete picture of heterogeneous genetic landscape of progressive disease. Mouse models provide a rational setting for studying the complementarity of CTC and ctDNA analyses in a controlled experimental environment.

There are also several limitations to the methods described here. The collected CTCs are fixed prior to identification, and so are not viable for culture or subsequent functional assays. Furthermore, RNA quality is compromised after fixation of cells, and single cell RNA analysis is not feasible for CTCs detected using this method. While this platform allows for inclusion of new markers, this step mandates assay development with a thorough inclusion of positive and negative controls, which is a limitation of all IF-based platforms. The fact that the *PIK3CA* mutation was not found in all the CTCs retrieved for sequence analysis highlights challenges in the interrogation of the genome of a single cell. We were only able to test for the mutation in those CTCs that provided a PCR amplicon spanning the mutation region after whole genome amplification. This could have been the result of poor overall WGA, absent coverage of this region in the WGA amplification reaction, or degradation of DNA within CTCs undergoing apoptosis and losing this region of the genome. Inefficiency and non-uniformity as well as inaccuracy of nucleic acid amplification at the one-cell level is a matter of intense current interest in the single-cell analysis research community [27, 28]. These issues will require improvement of molecular analysis protocols and optimization of methods in future studies.

Simultaneous evaluation of the phenotypic and genetic profiles of primary tumors, CTCs, disseminated tumor cells, and metastatic lesions is a major advantage of PDX models [9]. Such an analysis may allow better understanding of the metastatic process. Similarly, analysis using markers of survival and invasion/migration may provide insight into cellular plasticity of CTCs during transit to distant sites of metastasis. Recent reports have highlighted the importance of evaluating CTC clusters in the understanding of the metastatic process [29–31]. In this study using PDX models, we again detected CTC clusters (data not shown), which we had earlier observed with our IHC platform [9]. Future studies to interrogate the molecular and phenotypic differences between single and clustered CTCs may thus be possible using the cell retrieval capability of the RareCyte platform. The evaluation of CTCs in this study used four fluorescent channels to characterize cells. Recent advances in the RareCyte platform imaging technology have expanded the number of channels to six, allowing for up to three investigational biomarkers for CTC analysis [14]. Development of tailored assays using these additional biomarker channels will allow broader phenotypic characterization of CTCs to assess mesenchymal differentiation and tumor-specific oncogenic signaling.

## Conclusions

We demonstrate that the analysis of small volume blood samples from BC-PDX-bearing mice using the AccuCyte–CyteFinder system allows investigation of the role of CTCs in tumor biology and metastasis, independent of surface marker expression. Future real-time CTC analysis in PDX models for testing on-target effects of targeted drugs on signaling pathways and evaluating markers of treatment resistance may help provide a rational basis for the use of CTC analysis in guiding clinical treatment decisions.

## Additional file

Additional file 1 **Figure S1.** Linearity of spike-in CTC counts at low blood volumes. SK-BR-3 breast cancer cells were spiked into control mouse blood at approximately 500 cells/mL. Aliquots of 1000, 500, 250, 125 and 62.5 μL were made and processed according to the mouse blood protocol. Slides were analyzed after staining and imaging. CTC counts throughout the range of volumes tested were highly linear (R^2^ = 0.97). (PPTX 37 kb)

## Abbreviations

BC: Breast cancer
BC-PDX: Breast cancer patient-derived xenograft
CK: Pan-cytokeratin
CTC: Circulating tumor cell
HER2: Human epidermal growth factor receptor-2
IF: Immunofluorescence
IHC: Immunohistochemistry
RBC: Red blood cell
WBC: White blood cell
WGA: Whole genome amplification
