# Supplementary figures and images for: Circulating tumor cell investigation in breast cancer patient-derived xenograft models by automated immunofluorescence staining, image acquisition, and single cell retrieval and analysis
Source: BMC Cancer. 2019 Mar 12;19:220. doi: 10.1186/s12885-019-5382-1 (PMC6419430; doi:10.1186/s12885-019-5382-1)

## Slide 1
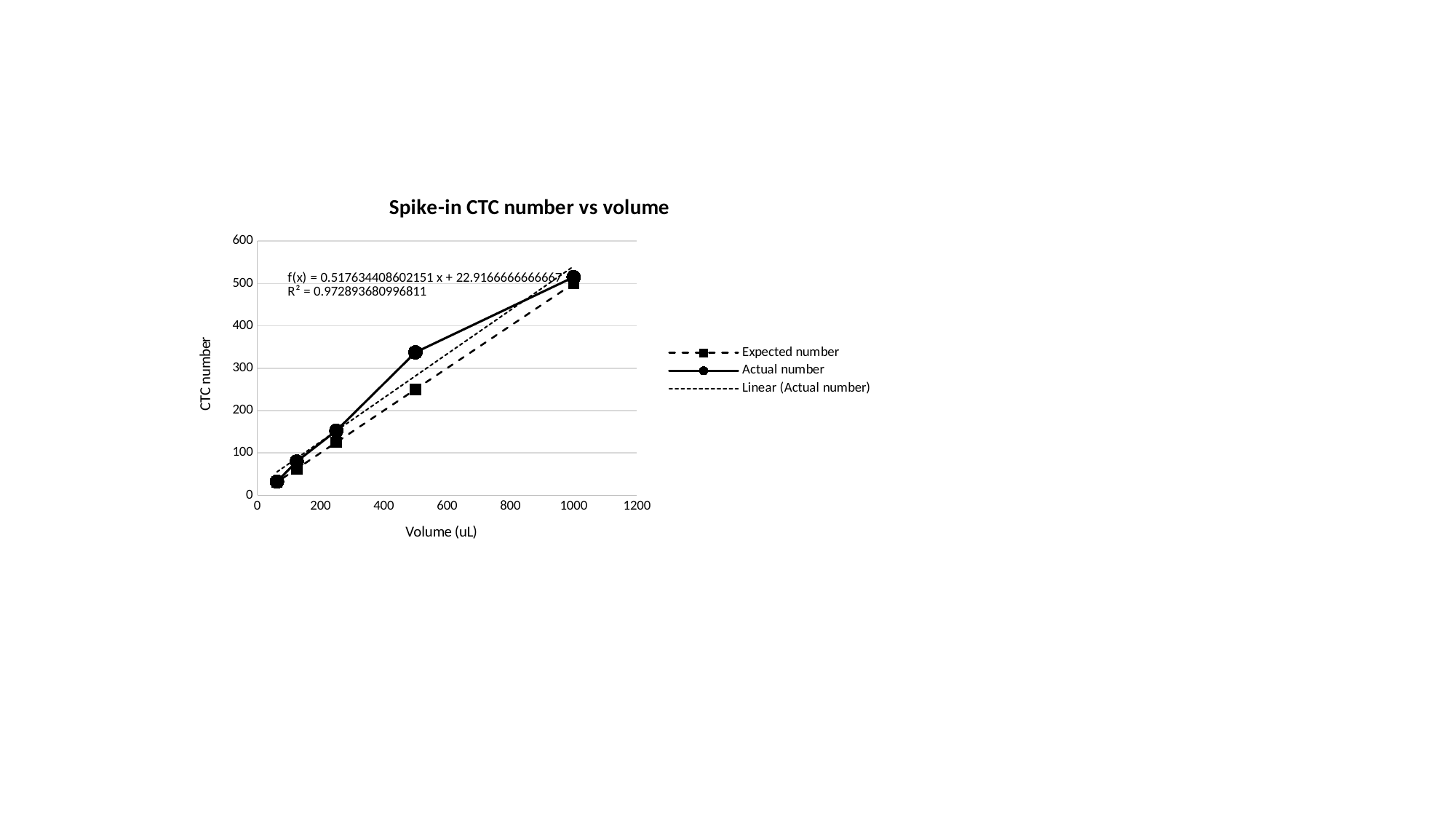

### Chart: Spike-in CTC number vs volume
| Category | | |
|---|---|---|

Supplement: Supplementary file 1 — Figure S1. Linearity of spike-in CTC counts at low blood volumes. SK-BR-3 breast cancer cells were spiked into control mouse blood at approximately 500 cells/mL. Aliquots of 1000, 500, 250, 125 and 62.5 μL were made and processed according to the mouse blood protocol. Slides were analyzed after staining and imaging. CTC counts throughout the range of volumes tested were highly linear (R2 = 0.97). (PPTX 37 kb) [file 12885_2019_5382_MOESM1_ESM.pptx]
